# Rural mental health strengths and social connectedness: Findings from the Australian National Study of Mental Health and Wellbeing

**DOI:** 10.1177/00048674261467613

**Published:** 2026-08-09

**Authors:** Annika Luebbe, Zoe Rutherford, Harvey Whiteford, Sandra Diminic

**Affiliations:** 1 Queensland Centre for Mental Health Research, Wacol, QLD, Australia; 2 The School of Public Health, The University of Queensland, Herston, QLD, Australia; 3 Toowoomba Regional Clinical Unit, Faculty of Health, Medicine and Behavioural Sciences, Rural Clinical School, The University of Queensland, South Toowoomba, QLD, Australia

**Keywords:** Rural, mental health, strengths, 2020–2022 National Study of Mental Health and Wellbeing, Rural Strengths Socioecological Framework

## Abstract

**Objective:**

This study aimed to describe rural data from the Australian National Study of Mental Health and Wellbeing survey (2020–2022) to assess strengths-based questions, differences by rurality, and factors associated with excellent social connectedness.

**Methods:**

Survey questions were mapped to the Rural Strengths Socioecological Framework. Descriptive and logistic analyses assessed rural adults’ use of strengths by remoteness and presence of affective/anxiety disorders in the past 12 months. Logistic regression explored factors associated with social connectedness.

**Results:**

Survey questions reflected some strengths within the Framework including self-management techniques, family and friend support, social connectedness, and health care personnel. However, questions were primarily framed using a deficit perspective and many strengths from the Framework (i.e. nature, rural norms, culture, and lay personnel) were absent. Most strengths did not differ significantly between levels of rurality, though making healthy diet choices (as a self-management technique) was significantly lower in remote areas. Adults without affective/anxiety disorders more often reported good social support and self-efficacy. Conversely, those with affective/anxiety disorders were more likely to self-manage and access consultations for mental health. Excellent social connectedness was associated with better mental health.

**Conclusion:**

The survey questions reflected limited strengths. Future surveys should include a broader range of strengths-based questions spanning the extent of the Rural Strengths Socioecological Framework. Healthy diet choices as a self-management tool in remote areas may need to be prioritised. Findings also suggest the need for targeted approaches to protect, improve, or leverage social connectedness for those with more severe illness in rural areas.

## Introduction

Despite extensive research and policy efforts (Batterham et al., 2020; Farmer et al., 2020; Hunter, 2007; Lawrence-Bourne et al., 2020; Lewis, 2020; Rosenberg and Roberts, 2020; Stanton et al., 2020; Wainer and Chesters, 2000), mental health disparities persist in rural Australia (Australian Institute of Health and Welfare, 2014; Elliott-Schmidt and Strong, 1997; Fuller et al., 2000; Gardiner et al., 2019; Green et al., 2012; Griffiths et al., 2009; Marshall and Dunstan, 2013; Wellbeing, Australian Institute of Health and Welfare, 2021). Solutions, although well-meaning, are not translating to improved outcomes and mental health inequities remain (Australian Government, 2023a; National Rural Health Alliance, 2021).

Approaches that are deficit-based (i.e. focus on shortcomings and aetiology of ill-health) and metrocentric (i.e. apply urban assumptions to rural contexts) minimise rural diversity, emphasise negative aspects of mental health, and prioritise clinical, often externally based resources to bridge the gap (Bourke et al., 2013; Malatzky and Bourke, 2016; Smith et al., 2008) and neglect alternate and non-clinical solutions (e.g. self-care) that rural populations could provide for themselves (Perkins et al., 2019). Rural communities continue to experience poorer mental health outcomes, access barriers, and service shortages, which to date have not improved despite substantial policy focus and funding (Australian Government, 2023a). Rather than viewing rural communities as solely and inherently problematic, research, policy development, and service planning should also acknowledge pre-existing and naturally occurring rural strengths – assets, resources, or protective factors positively associated with mental health (Bourke et al., 2010; Wakerman, 2008).

Our systematic review of Australian and North American literature identified an array of strengths that exist in rural communities across the extent of our Rural Strengths Socioecological Framework (RSSF) (Luebbe et al., 2024) (natural environment, intrapersonal, interpersonal, community, and personnel). These rural strengths already support mental health in diverse ways including participation in Men’s Sheds linked to fewer self-reported symptoms of depression (Culph et al., 2015), spirituality (beliefs and behaviours) negatively associated with depression (Yoon and Othelia Lee, 2004), social and community resources positively impacting women’s mental health (Panazzola and Leipert, 2013), greater social capital associated with less distress (Berry, 2009), and community supports related to better mental health (Stain et al., 2008). Underpinning these identified strengths is social connectedness, a well-established protective factor against mental illness (Wickramaratne et al., 2022), and likely an underlying multidimensional construct associated with changes in depressive symptoms (Galloway, 2013). To date, limited research has explored the causality of the benefits of these strengths; however, correlations with improved mental well-being have been cited (Luebbe et al., 2025).

Rural communities have greater social capital and social cohesion compared to metropolitan counterparts (Ziersch et al., 2009). An increase in social capital has been associated with reduced prevalence of mental disorders, acting as a protective factor against mental illness by decreasing risk, and improving recovery from mental illness (Berry and Welsh, 2010; Henderson and Whiteford, 2003). The collective strength of local communities, their sense of belonging, and social support can foster community and individual resilience and these have also been identified as beneficial to mental health (Allan, 2010; Ziersch et al., 2009). Further investigation is required to understand what makes social cohesion contextually appropriate, for which groups social connectedness is or is not strong, and how it can be effectively leveraged (for those socially connected) or improved (for those not socially connected) to prevent mental ill-health.

For those who cannot access services in rural areas and suffering with mental health problems, leveraging individual strengths is also a valuable resource. In 2010, Olesen et al. (2010) investigated the prevalence of self-management and formal service use for common mental disorders in Australia from the 2007 National Survey of Mental Health and Wellbeing (Australian Bureau of Statistics [ABS], 2008). They found self-management techniques used with and without adjunct formal services were prevalent throughout Australia regardless of participants having diagnosable affective or anxiety disorders (Olesen et al., 2010). As such, supporting self-management skills in resource-poor locations may be a viable treatment option, as has been shown in other health research (Nelson et al., 2022).

Despite their relevance, rural strengths remain under-researched and under-utilised in mental health planning due to a limited evidence base and clinical bias in current approaches (Luebbe et al., 2025). Therefore, understanding and producing stronger evidence for local rural assets, resources, or strengths may be critical in reshaping the rural health system. Research to date has also not explored how strengths differ by rurality, despite indications that mental health, community, and health system organisation are different. Similarly, it is unknown whether the preference of strengths varies depending on the presence of mental disorders. Like suicide rates (Australian Government, 2023b) and access to services (Amos et al., 2023), strengths may also differ by rurality. Investigating strengths used by rural Australians will enhance understanding of strengths, how they can be used, and how strengths-based data collection and evidence can be improved.

This study drew on nationally representative population data from the 2020–2022 National Study of Mental Health and Wellbeing (NSMHW) (ABS, 2023a) to explore how rural adult Australians across different remoteness areas, with and without mental disorders, identify and use strengths. Specifically, the aims were to examine (1) whether a national survey captured rural strengths identified in the RSSF, (2) differences in strength identification or use by (a) remoteness and (b) the presence of common mental disorders (i.e. anxiety and affective disorders), and (3) sociodemographic characteristics associated with higher social connectedness.

## Methods

### NSMHW survey – data collection

This study analysed ABS NSMHW survey data (2020–2022) (ABS, 2023a). The survey collected data on the prevalence of mental disorders including self-management of mental health problems, social connectedness, family and friend support, and service utilisation. A two-stage random sample of Australian households was conducted, with one adult per household randomly selected. Data were collected in two cohorts: December 2020 to July 2021 (5554 fully responding households) and December 2021 to October 2022 (10,339 fully responding households). The combined cohorts totalled 15,893 fully responding households (52% response rate). Survey participants were usual residents in Australia aged 16–85 who were living in private dwellings (non-private dwellings excluded, e.g., hospitals, hotels, nursing homes, homelessness), in all states and territories, from urban (*n* = 12,040) and rural areas (inner regional, outer regional, and remote) (*n* = 3853) excluding very remote parts of Australia and discrete Aboriginal and Torres Strait Islander communities (ABS, 2023a).

### NSMHW survey – data content

The World Mental Health Composite International Diagnostic Interview (WMH-CIDI) was scored to determine lifetime and 12-month diagnostic criteria for each mental disorder (ABS, 2024). Interviews used structured WMH-CIDI 3.0 protocols administered by trained ABS interviewers following standardised training procedures. Interviewers did not probe beyond structured questions. For the purposes of this study, we focused on anxiety (e.g. generalised anxiety disorder, post-traumatic stress disorder [PTSD]) and affective (e.g. depression, bipolar) disorders meeting diagnostic criteria in the last 12 months. Alcohol and drug use disorders, although prevalent and often comorbid, were outside the scope of this analysis. Disorders were classified by severity (mild, moderate, severe) using the Diagnostic and Statistical Manual of Mental Disorders, 4th edition (*DSM*-IV) (American Psychiatric Association, 2000) Australian severity version, based on their diagnosis and overall level of functional impairment (ABS, 2024).

Service utilisation was identified by consultations with mental health service providers during the past 12 months. Social connectedness was self-rated on a 5-point Likert-type scale (poor, fair, good, very good, to excellent) based on the question ‘sense of being part of a group or community’. Friend and family support was collected across several yes/no questions including ‘whether [they] did any activities with family or friends in the last four weeks’. A 5-point Likert-type scale measured ‘ability to get support from family or friends when needed’ (poor, fair, good, very good, to excellent). Self-management was identified by participants responding to the question ‘type of self-management strategies used for mental health in the past 12 months’. Eleven multiple response options included seeking support from family or friends, practising meditation, and reducing and/or using alcohol and drugs. Most of these identified strategies of are known to be beneficial in managing mental health including increasing physical activity, practising meditation, and avoiding alcohol and drugs (Jorm et al., 2002, 2004). As this study is concerned with identifying and analysing strengths, and in line with Olesen’s study of self-management questions (Olesen et al., 2010), drug and alcohol use was not considered a potentially beneficial self-management strategy. Other self-management strategies including family/friends and faith/spirituality appeared as possible responses for several questions related to preferring to self-manage ‘main reason for preferring to manage own mental health’ where the participant identified a mental health problem and receiving insufficient support.

Other non-identifiable demographic information was collected and used within the analysis including age, sex, rurality, household income, and Indigenous status. The NSMHW classified rurality using ARIA+, Australian Statistical Geography Standard (ASGS) 2016.

### Data analysis

To identify strengths elicited throughout the survey, all survey questions were systematically reviewed and classified into levels and categories of the RSSF (Luebbe et al., 2024) (see Table 1). To identify use of strengths by rural populations, the survey questions mapped to the Framework were descriptively analysed by level of rurality (inner regional, outer regional, remote) and for those with and without 12-month affective/anxiety disorders. The remote population in this survey was small (*n* = 87) so to protect the anonymity of survey participants, remote and outer regional populations were collapsed where necessary (i.e. in the service utilisation analysis). To investigate differences in strengths between geographical locations or the presence of common mental disorders, chi-square tests compared use of strengths across the nominal variables, rurality level and 12-month disorders.

**Table 1.** Mapping NSMHW survey questions to the Rural Strengths Socioecological Framework (RSSF).

Socioecological level	Strength categories	Strength sub-categories	Definition	NSMHW questions (Summarised/combined + population asked)
Natural Environment	Admiration and Connection	Natural beauty	Engaging with or exposure to nature in some capacity that benefits mental well-being	X^a^
		Connection to Country, place attachment		X
	Interaction	Caring-for-country-practice		X
		Engaging, gardening, animals		X
Intrapersonal Level	Knowledge	Access to information	Exposure or access to mental health information and mental health literacy	X
		Mental health awareness, education		X
		Passing of knowledge		X
		Self-efficacy	Confidence to speak up in community or for own mental health	SC6OUT^b^: All persons asked if they had the confidence to have a say about important issues
	Attitudes	Sense of belonging, identity, purpose	Attitudes such as feeling safe in the community, sense of belonging that enhance mental well-being	SC7OUT: All persons asked if they felt a sense of being part of a community or group
		Belief in oneself, self-awareness		X
		Trust in provider, social trust		X
		Safety		X
	Personal Attributes	Optimism	Personal attributes that may allow someone to manage mental health problems safely and positively and be beneficial to mental well-being	X
		Resilience, strength of character		X
		Self-esteem		X
		Coping skills, self-reliance, self-help, self-management		SMSPS12: All persons asked what type of self-management strategies they used for mental health – improved diet, meditation/yoga/relaxation, organised home, thinking positively, achievable goals, exercise etc. SR140B-SR169B, RNMINFO, RNMMEDS, RNMTHPY, RNMHOUS, RNMWORK, RNMCARE, RSMPPL: People who said they needed help but did not receive help, or not enough help, or did not seek help (information/medication/counselling/money, housing/work/help to look after home/meeting people) for mental health were asked if the main reason for not getting enough was because they preferred to self-manage themselves specifically for mental health information/medications/counselling/housing and money problems/ability to look after self/ability to work/meeting new people SR140C-SR169C, RSMINFO, RSMMEDS, RSMTHPY, RSMHOUS, RSMWORK, RSMCARE, RSMPPL: People who said they needed help but did not receive enough help and those that did not seek help (information/medication/counselling/money, housing/work/help to look after home/meeting people) and preferred to manage themselves were asked what the main reason for their self-managing preference was
		Positive thinking, meaning-seeking, positive attitude to help-seeking		SMSPS12: All persons asked what type of self-management strategies they used for mental health – Practised thinking positively and setting achievable goals
		Appreciation and hope, acceptance, inner peace		X
	Faith and Cultural Beliefs	Religiosity, spirituality	Maintenance or engagement with religious or cultural beliefs beneficial to mental well-being	SR140C-SR169C, RSMINFO, RSMMEDS, RSTHPY, RSMHOUS, RSMWORK, RSMCARE, RSMPPL: People who preferred to self-manage and did not receive enough or any information/medication/counselling/help with housing/help with work/help looking after home/help to meet people were asked if their main reason for self-managing was because they relied on faith and spirituality
		Cultural spiritual beliefs		X
		Cultural identity, connection		X
Interpersonal Level	Family and Friends	Family and friend support	Access to or involvement in informal connection from family and friends that is beneficial for mental health, or who can act as a support network, refer onto appropriate care etc.	SC1OUT, SC5OUT: All persons asked whether they did any activities with family or friends in the last 4 weeks (yes/no) and their ability to get support from friends and family when needed SMSPS12: All persons asked if they sought support from friends and family as a type of self-management strategy for mental health SR140C-SR169C, RSMINFO, RSMMEDS, RSMTHPY, RSMHOUS, RSMCARE, RSMPPL: People who said they did not seek help but needed it/did not receive enough help and preferred to self-manage mental health/mental health information/medication/counselling/money/ability to work/look after self and home/meeting people for support were asked if the main reason for preferring to self-manage was because they relied on friends and family
	Peers	Peer, social, community support	Access to or involvement in informal connection from peers, neighbours that is beneficial for mental health, or who can act as a support network, refer onto appropriate care etc	SR159C, RSMPPL, SR169C, SR158B, SR158C, SR159B, RNMPPL, SR169B: People who did not receive help to meet people but needed it who preferred to self-manage were asked why they preferred to self-manage
		Neighbours		X
		Co-workers		X
Community Level	Norms and Standards	Local/rural norms	Alternative and flexible care solutions that may be a result of lacking resources or rural cultural norms such as using what is available, coordinating with other services, providers travelling to see consumers etc. that assist in managing mental health	X
		Gemeinschaft characteristics		X
		Flexible, adaptable, innovative, informal delivery		X
		Task sharing/shifting, coordination, cooperation, interdisciplinary collaboration		X
		Indigenous lore, customs		X
	Culture	Cultural tradition, activities, practice	Engagement or involvement in cultural practices beneficial to mental well-being	X
		Cultural retention		X
	Social Networks	Social and community connectedness	Engagement in and socialisation with other community members in varying capacities that are beneficial to mental health that can enhance a sense of belonging and grow social connection	SC7OUT: All persons asked if they felt a sense of being part of a community or group
		Community group		SC2OUT: All persons asked if they did any activities with community groups or clubs
		Arts group		X
		Sports teams/clubs/activities (football, netball, curling, fishing)		X
		Men’s shed		X
		Local events, volunteering		SC3OUT: All persons asked if they participated in any organised volunteer work
		Church	Facilities or amenities providing mental well-being support/information and/or a physical useable gathering space	X
		Schools, universities		X
		Community centre, banks, Drs office, clubs		SSS2OUT: All persons asked if they sought assistance from social support services including housing, legal aid, youth support, family/parenting support
		Provider lived experience with mental health	Mental health provider having lived experience of mental illness beneficial to providing relevant care	X
		Provider lived experience with/appreciation of rural life/culture, cultural competence, community involvement	Mental health provider having rural or local understanding and/or community involvement beneficial to providing relevant care	X
Personnel Level	Lay Community Members	Community member	Local rural community members who are part of the community and have established place in the community who can identify/screen for poor mental well-being and provide informal support, refer to appropriate care	X
		Clergy		X
		School teacher		X
		Rural extension agent		X
		Rural financial counsellor		X
		Elders, indigenous, emergency services		X
		Sports coach, leader, instructor		X
	Health Care Personnel	Other/General health care professional	Local health care providers who can identify/screen for poor well-being in patients and provide support and refer to appropriate care, may be trusted member of community and know mental health service providers to refer on	MHOGEN12, MHCONS: All persons asked whether they accessed/had a consultation with any health professional or ‘other’ health professional for mental health in the last 12 months
		Nurse		X
		Pharmacist		X
		General Practitioner, primary health care provider		MHODR12, MHGP12: All persons asked whether they had had a consultation with a doctor, specialist doctor or surgeon for mental health in the last 12 months
		Community health worker, health promoter		X
		Peer support worker		X
		Mental health nurse	Mental health trained clinicians/carers who can identify/screen for poor well-being and provide appropriate care	MHMHN12: All persons asked whether they had a consultation with a mental health nurse in the last 12 months
		Psychologist, psychiatrist, mental health practitioner		MHPSYO12, MHPSYI12, MHOMH12, MHMHPR12: All persons asked whether they had had a consultation with a psychologist, psychiatrist, ‘any’ or ‘other’ mental health professional over the last 12 months

a X reflects having no questions related to these strengths asked in the survey.

b Codes refer to SAS codes used in survey, see 2020–2022 NSMHW data citation (ABS, 2023a) in references for more detail.

A multivariate logistic regression analysis was run to explore sociodemographic characteristics associated with excellent social connectedness (excellent vs very good/good/fair/poor) for rural populations (inner regional/outer regional/remote combined). To assess correlates, social connectedness was stratified across sociodemographic characteristics including age, sex, *DSM*-IV mental disorder severity, highest level of education, marital status, socioeconomic disadvantage (quintiles based on the ABS area-level Index of Relative Socioeconomic Disadvantage (IRSD); (ABS, 2023b) household composition, and household income.

To assess for multicollinearity, pairwise comparisons (Cramer’s *V*) and variation inflation factors (VIFs) were run, outlining high multicollinearity between comorbidity of affective/anxiety disorders and disorder severity. Therefore, comorbidity was removed from the regression model. Some variables of interest (self-management, social connectedness, education level, income) had missing data often labelled as not stated, not known or not applicable. To determine whether missing data should be removed or not, the rural population was compared for those with missing data vs not missing data and there were no significant differences in age (χ^2^ = 73.2, *p* = 0.115) or sex (χ^2^ = 0.6, *p* = 0.756). There was a significant difference for social connectedness with 27.9% of people with non-missing data and 34.9% of people with missing data having excellent social connectedness (χ^2^ = 28.5, *p* = 0.039). Rural survey participants with missing data (*n* = 296, 7.68% of rural population) were excluded from the regression analysis.

An ethics exemption for this study was approved by the University of Queensland (2024/HE000067). Stata 18 (StataCorp, 2023) software was used to conduct all analyses of de-identified survey data within the ABS DataLab secure, remote access platform. Survey weights were applied to represent the overall Australian population in calculating estimates and jackknife methodology calculated 95% confidence intervals (CIs). The significance threshold for this study was *p* < 0.05.

## Results

### Survey questions reflecting strengths

Table 1 presents the NSMHW survey questions and their alignment with strengths in the RSSF (Luebbe et al., 2024); within every socioecological level are strength categories and sub-categories that contextualise what a strength is and where it may exist in the rural community. The distribution of strengths reflected in survey questions was mixed. Some Framework levels did not have any corresponding survey questions such as the natural environment, while other strength categories and sub-categories were not reflected including local norms and standards, culture, and lay community members. Reflected strengths included intrapersonal strengths (self-efficacy, spirituality, self-management), interpersonal (especially friend and family support), community (community groups, volunteering), and health care personnel (GPs, nurses, psychologists etc.).

The ‘family and friends’ sub-categories were combined in this version of the Framework as the survey items did not distinguish between friend and family support. An extra sub-category was added in the Personnel level labelled ‘Other/General health care professional’ as some survey questions asked more broadly about participants seeking support from ‘any’ health care provider.

Most of the identified survey questions in Table 1 were asked of everyone. However, some questions were only asked of a particular group of people and thus may not provide an accurate reflection of the whole rural population. For example, some self-management questions were only asked of a smaller subsample of people who met specific criteria, e.g. having a mental health issue and not getting enough help because they preferred to self-manage.

### Strengths by level of rurality

Table 2 shows the use of self-management strategies, social support services, social connectedness and mental health consultations by rurality (national average vs inner regional vs outer regional vs remote). Apart from healthy diet choices, the use or identification of strengths did not differ significantly depending on remoteness.

**Table 2.** Adults’ use of strengths and self-management for mental health by rurality of residence.

Strengths: Management strategies identified in survey questions	Australia (total) *n* = 15,893	Inner regional *n* = 2626	Outer regional *n* = 1140	Remote *n* = 87	Chi square (inner regional vs outer regional vs remote) *n* = 3853
	Total % (95% CI)	Total % (95% CI)	Total % (95% CI)	Total % (95% CI)	χ^2^ (*p*)
*Intrapersonal level*					
Self-efficacy: Confidence to have a say about important issues (good/very good/excellent)	92.8% (92.2%, 93.4%)	93.4% (92.0%, 94.5%)	91.0% (88.2%, 93.1%)	88.1% (69.0%, 96.1%)	χ^2^(4) = 14.3 (*p* = 0.157)
Self-management overall^a,b^	62.2% (61.1%, 63.2%)	60.7% (58.0%, 63.4%)	58.3% (53.6%, 62.9%)	49.4% (36.0%, 62.8%)	χ^2^(2) = 6.1 (*p* = 0.234)
*Self-management strategies used*					
Increased exercise/physical activity	37.3% (36.3%, 38.4%)	33.3% (30.8%, 36.0%)	33.1% (29.0%, 37.4%)	26.7% (17.1%, 39.1%)	χ^2^(2) = 1.8 (*p* = 0.604)
Healthy diet choices^c^	31.3% (30.2%, 32.3%)	28.6% (26.6%, 30.7%)	32.1% (27.7%, 36.8%)	16.8% (9.2%, 28.7%)	χ^2^(2) = 12.0 (*p* = 0.036)
Practised positive thinking/achievable goals	29.1% (28.1%, 30.0%)	28.3% (25.9%, 30.8%)	26.8% (22.8%, 31.3%)	22.3% (15.44, 31.2%)	χ^2^(2) = 2.2 (*p* = 0.488)
Did more enjoyable things	28.9% (27.9%, 29.9%)	28.4% (26.2%, 30.8%)	25.6% (22.7%, 28.7%)	18.9% (11.7%, 29.1%)	χ^2^(2) = 6.7 (*p* = 0.083)
Better organised home, life, other things	26.9% (26.1%, 27.8%)	24.5% (22.0%, 27.2%)	24.1% (20.5%, 28.2%)	20.1% (11.2%, 33.3%)	χ^2^(2) = 1.0 (*p* = 0.761)
Practised meditation/mindfulness/yoga	23.9% (23.1%, 24.7%)	20.1% (18.2%, 22.1%)	19.0% (15.7%, 22.7%)	12.0% (6.2%, 22.1%)	χ^2^(2) = 4.1 (*p* = 0.316)
Those with mental health problem who did not receive enough help and preferred to self-manage^d^	4.4% (4.1%, 4.8%)	4.0% (3.1%, 5.0%)	5.7% (4.2%, 7.6%)	Not reported due to small underlying raw numbers	χ^2^(2) = 6.5 (*p* = 0.087)
Relied on Faith and spirituality	0.2% (0.1%, 0.2%)	–	–	–	–
*Interpersonal level*					
Ability to get support from family/friends when needed (good/very good/excellent)	93.7% (93.2%, 94.2%)	93.9% (92.6%, 95.0%)	93.2% (90.9%, 95.0%)	98.1% (90.5%, 99.7%)	χ^2^(4) = 4.5 (*p* = 0.671)
Any activities with family/friends in last 4 weeks	93.2% (92.6%, 93.8%)	91.7% (90.1%, 93.1%)	89.7% (86.7%, 96.7%)	92.7% (84.5%, 96.7%)	χ^2^(4) = 5.4 (*p* = 0.612)
Sought support from family and friends	23.2% (22.3%, 24.1%)	22.4% (20.3%, 24.6%)	20.9% (16.9%, 25.5%)	23.3% (15.2%, 33.8%)	χ^2^(2) = 1.2 (*p* = 0.672)
*Community level*					
Social connectedness: Sense of being part of a group/community (good/very good/excellent)^b^	86.7% (85.9%, 87.5%)	85.1% (82.4%, 87.5%)	83.6% (79.8%, 86.8%)	88.5% (69.7%, 96.3%)	χ^2^(4) = 2.8 (*p* = 0.847)
Any activities with community groups/clubs in last 4 weeks	37.7% (36.7%, 38.7%)	37.7% (35.3%, 40.1%)	41.1% (36.0%, 46.3%)	43.6% (22.4%, 67.4%)	χ^2^(4) = 5.8 (*p* = 0.756)
Any organised volunteer work in last 4 weeks	13.9% (13.2%, 14.6%)	16.3% (14.2%, 18.6%)	17.6% (14.7%, 20.9%)	26.0% (11.5%, 48.6%)	χ^2^(4) = 7.2 (*p* = 0.619)
Used social support services^e^	9.3 (8.6%, 10.0%)	8.0% (6.5%, 9.5%)	11.0% (8.4%, 14.3%)	Not reported due to small underlying raw numbers	χ^2^(2) = 10.3 (*p* = 0.123)
*Personnel level*					
Service utilisation: mental health consultations in last 12 months:^f^					
Any health professional	17.3% (16.5%, 18.1%)	18.8% (16.7%, 21.0%)	15.3% (12.5%, 20.9%)		χ^2^(4) = 8.0 (*p* = 0.515)
General practitioner	12.4% (11.7%, 13.1%)	13.8% (12.1%, 15.8%)	11.5% (9.2%, 14.3%)		χ^2^(4) = 6.7 (*p* = 0.3725)
Any mental health professional	11.9% (11.3%, 12.5%)	11.9% (10.2%, 13.7%)	9.8% (7.9%, 12.1%)		χ^2^(4) = 6.8 (*p* = 0.582)
Psychologist	7.8% (7.3%, 8.3%)	6.1% (5.1%, 7.3%)	6.1% (4.6%, 8.1%)		χ^2^(4) = 0.3 (*p* = 0.970)
Psychiatrist	3.3% (3.0%, 3.6%)	3.6% (2.6%, 5.0%)	2.0% (1.3%, 3.1%)		χ^2^(4) = 7.7 (*p* = 0.369)
Other mental health professional^g^	3.6% (3.2%, 4.0%)	4.8% (3.6%, 6.2%)	3.9% (2.7%, 5.7%)		χ^2^(4) = 4.6 (*p* = 0.695)

a Anyone that endorsed using self-management strategies for mental health in the past 12 months.

b Missing data removed from variable: self-management overall (*n* = 3), social connectedness (*n* = 6).

c Combined variables that endorsed healthy choices: taken non-prescription medicines, vitamins, supplements or herbal remedies, changed diet, cut out alcohol and drugs.

d Those with self-identified mental health conditions or issues who reported that they did not receive enough/any help for their mental health because they preferred to self-manage.

e Combines social support service use in the past 12 months variables including housing/accommodation, legal aid, job search/employment, youth, family/parenting, financial.

f Outer regional and regional and remote service utilisation combined due to small underlying scores.

g Including mental health nurse.

#### Intrapersonal

Around 50–60% of rural populations reported self-managing their mental health. The most frequent strategy was physical activity (27.7–33.3%) followed by healthy diet choices (16.8–32.1%), which was a culmination of responses including taking vitamins, supplements or herbal remedies, changing diet and cutting out alcohol and drugs. Healthy diet choices had significant differences across rurality with lower self-management in remote areas (28.6% inner regional, 32.1% outer regional, 16.8% remote, χ^2^(2) = 12.0, *n* = 3853, *p* = 0.036). Those with mental health problems who did not receive enough help (and preferred to self-manage) ranged from 4.0% in inner regional to 5.7% in outer regional. However, the remote area population could not be reported as the raw sample size was too small. A small percentage of survey participants (0.2%) who reported that they did not receive enough help due to preferring to self-manage identified faith and spirituality as their chosen approach. Due to the small size, this could not be broken down by rurality. This was the only question related to faith and spiritual practices, so it is unknown whether the broader population also uses this self-management strategy. Participants rated their confidence to have a say about important issues in their community ranging from 88.1% to 93.4%.

#### Interpersonal

In the interpersonal level, the most common strengths were the ability to get support from family and friends (93.2–98.1%), followed by activities with family and friends (91.7–92.7%), then seeking out support from family and friends (20.9–23.3%).

#### Community

Good social connectedness was the most common strength identified in the community level (83.6–88.5%), followed by activities with community groups or clubs (37.7–43.6%). Volunteer work, although not significantly different, was highest in remote areas (16.3% inner regional, 17.6% outer regional, 26.0% remote). Social support service use was lowest at 8.0–11.0% in rural areas.

#### Personnel

In the personnel level, 15.3–18.8% of rural people had a mental health consultation with any health professional. This was most commonly GPs (11.5–13.8%) and least commonly psychiatrists (2.0–3.6%).

### Strengths by those with and without disorders

Table 3 describes similar variables to Table 2 but stratified against collapsed levels of rurality (national average vs inner regional vs combined outer regional and remote) across those with and without 12-month anxiety and/or affective disorders. Across almost all variables, there were significant differences in use of strengths for those with and without disorders excluding activities with friends and family, and volunteering. The patterns of strength identification or use by disorder were similar in both inner regional and outer regional/remote areas.

**Table 3.** Adults’ use of strengths and self-management for mental health for those with and without 12-month disorders by level of rurality.

	With 12-month affective/anxiety disorders % (95% CI)			Without 12-month affective/anxiety disorders % (95% CI)			Chi square (disorder vs no disorder in inner regional, outer regional, and remote) (*n* = 3853)
Strengths: Management strategies identified in survey questions	Australia (total) *n* = 15,893	Inner Regional *n* = 2626	Outer Regional and Remote *n* = 1227	Australia (total) *n* = 15,893	Inner Regional *n* = 2626	Outer Regional and Remote *n* = 1227	χ^2^ (df) (*p*)
*Intrapersonal level*							
Self-efficacy: Confidence to have a say about important issues (good/very good/excellent)	81.4% (79.3%, 83.3%)	81.5% (75.4%, 86.3%)	77.1% (68.5%, 83.9%)	95.4% (94.8%, 96.0%)	96.2% (95.0%, 97.1%)	93.7% (91.4%, 95.4%)	χ^2^(2) = 830.6 (*p* < 0.001)
Self-management overall^a,b^	85.7% (84.0%, 87.3%)	86.1% (82.7%, 89.0%)	84.3% (75.2%, 90.5%)	56.8% (55.6%, 58.0%)	54.6% (51.3%, 57.8%)	51.9% (46.9%, 57.0%)	χ^2^(1) = 1021.6 (*p* < 0.001)
*Self-management strategies used:*							
Healthy diet choices^c^	52.0% (49.6%, 54.3%)	52.0% (46.6%, 57.4%)	49.2% (39.3%, 59.2%)	26.5% (25.4%, 27.6%)	23.0% (20.7%, 25.3%)	27.0% (22.7%, 31.8%)	χ^2^(1) = 840.8 (*p* < 0.001)
Increased exercise/physical activity	51.3% (49.0%, 53.8%)	47.2% (41.8%, 52.6%)	50.0% (37.7%, 62.3%)	34.1% (33.0%, 35.2%)	30.0% (27.1%, 33.1%)	28.9% (25.0%, 33.0%)	χ^2^(1) = 373.6 (*p* < 0.001)
Practised positive thinking/achievable goals	48.9% (46.5%, 51.3%)	52.7% (46.7%, 58.7%)	49.6% (40.5%, 58.8%)	24.5% (23.5%, 25.5%)	22.4% (19.9%, 25.1%)	21.5% (17.8%, 25.8%)	χ^2^(1) = 1064.7 (*p* < 0.001)
Did more enjoyable things	43.6% (41.3%, 45.9%)	43.7% (37.3%, 50.2%)	41.4% (33.3%, 50.0%)	25.5% (24.5%, 26.5%)	24.8% (22.2%, 27.6%)	21.6% (18.5%, 25.0%)	χ^2^(1) = 451.9 (*p* < 0.001)
Better organised home, life, other things	43.2% (41.5%, 45.5%)	42.5% (36.4%, 48.9%)	39.3% (32.2%, 46.9%)	23.2% (22.3%, 24.1%)	20.2% (17.7%, 23.0%)	20.5% (17.2%, 24.2%)	χ^2^(1) = 594.0 (*p* < 0.001)
Practised meditation/mindfulness/yoga	40.3% (37.8%, 42.9%)	38.0% (31.0%, 45.5%)	26.4% (19.1%, 35.3%)	20.1% (19.1%, 21.1%)	15.8% (14.0%, 17.8%)	16.7% (13.2%, 21.0%)	χ^2^(1) = 513.2 (*p* < 0.001)
Those with mental health problem who did not receive enough help and preferred to self-manage^d^	14.5% (13.1%, 16.1%)	11.8% (8.8%, 15.7%)	20.1% (14.4%, 27.2%)	2.1% (1.9%, 2.4%)	2.1% (1.3%, 3.3%)	2.3% (1.3%, 4.0%)	χ^2^(1) = 852.7 (*p* < 0.001)
*Interpersonal level*							
Ability to get support from family/friends when needed (good/very good/excellent)	83.2% (81.4%, 84.9%)	85.0% (80.0%, 89.0%)	77.5% (67.7%, 84.9%)	96.1% (95.7, 96.5%)	96.0% (94.8%, 97.0%)	97.0% (95.2%, 98.2%)	χ^2^(2) = 788.1 (*p* < 0.001)
Any activities with family/friends in last 4 weeks	91.9% (90.5%, 93.1%)	89.5% (84.8%, 92.9%)	91.0% (86.0%, 94.3%)	93.5% (92.8%, 94.2%)	92.3% (90.3%, 93.9%)	89.7% (86.7%, 92.1%)	χ^2^(2) = 12.2 (*p* < 0.348)
Sought support from family and friends	42.5% (40.3%, 44.7%)	39.4% (33.1%, 46.0%)	42.1% (32.7%, 52.2%)	18.8% (17.9%, 19.7%)	18.3% (16.0%, 20.8%)	16.5% (12.9%, 20.8%)	χ^2^(1) = 723.2 (*p* < 0.001)
*Community level*							
Social connectedness: Sense of being part of a group/community (good/very good/excellent)^b^	67.4% (65.1%, 69.7%)	65.1% (57.7%, 72.0%)	60.5% (49.3%, 70.7%)	91.1% (90.4%, 91.8%)	89.9% (88.0%, 91.5%)	89.0% (85.5%, 91.8%)	χ^2^(2) = 1260.5 (*p* < 0.001)
Any activities with community groups/clubs in last 4 weeks	31.7% (29.3%, 34.2%)	31.3% (26.5%, 36.6%)	37.8% (29.6%, 46.7%)	39.1% (38.0%, 40.2%)	39.2% (36.6%, 41.9%)	42.0% (36.7%, 47.5%)	χ^2^(2) = 49.1 (*p* < 0.010)
Any organised volunteer work in last 4 weeks	11.9% (10.5%, 13.3%)	15.6% (11.8%, 20.3%)	11.3% (7.1%, 17.3%)	14.4% (13.6%, 15.2%)	16.4% (14.2%, 18.9%)	19.7% (16.5%, 23.4%)	χ^2^(2) = 25.6 (*p* = 0.094)
Used social support services^e^	16.0% (14.3%, 18.0%)	13.3% (9.5%, 18.1%)	20.0% (13.7%, 27.9%)	7.7% (7.0%, 8.4%)	6.6% (5.4%, 8.1%)	8.6% (6.2%, 12.0%)	χ^2^(1) = 195.1 (*p* < 0.001)
*Personnel level*							
Service utilisation: mental health consultations in last 12 months:							
Any health professional	48.7% (46.1%, 51.3%)	50.3% (44.8%, 55.9%)	42.3% (32.8%, 52.4%)	10.1% (9.4%, 10.8%)	11.2% (9.5%, 13.1%)	9.5% (7.1%, 12.6%)	χ^2^(2) = 2320.3 (*p* < 0.001)
General practitioner	39.0% (36.5%, 41.5%)	42.2% (36.7%, 48.0%)	36.4% (27.6%, 46.3%)	6.2% (5.7%, 6.9%)	7.0% (5.7%, 8.6%)	6.1% (4.3%, 8.6%)	χ^2^(2) = 2524.7 (*p* < 0.001)
Any mental health professional	33.4% (31.2%, 35.6%)	31.3% (26.4%, 36.6%)	29.6% (22.4%, 38.1%)	7.0% (6.4%, 7.6%)	7.2% (5.7%, 9.2%)	5.5% (4.0%, 7.5%)	χ^2^(2) = 1463.5 (*p* < 0.001)
Psychologist	22.7% (21.0%, 24.6%)	17.9% (14.2%, 22.2%)	19.5% (13.0%, 28.0%)	4.3% (3.9%, 4.8%)	3.3% (2.5%, 4.3%)	3.2% (2.1%, 4.8%)	χ^2^(2) = 963.0 (*p* < 0.001)
Psychiatrist	10.8% (9.6%, 12.2%)	10.7% (8.1%, 14.1%)	7.0% (3.9%, 12.2%)	1.6% (1.3%, 1.9%)	1.9% (1.0%, 3.6%)	1.0% (0.5%, 1.9%)	χ^2^(2) = 591.5 (*p* < 0.001)
Other mental health professional^f^	10.8% (9.5%, 12.4%)	12.6% (8.8% 17.6%)	12.7% (7.3%, 21.0%)	1.93% (1.6%, 2.3%)	2.9% (1.9%, 4.2%)	2.1% (1.2%, 3.4%)	χ^2^(2) = 604.1 (*p* = < 0.001)

a Anyone that endorsed self-management.

b Missing data removed from variable: self-management overall (*n* = 3), social connectedness (*n* = 6).

c Combined variables that endorsed healthy choices: taken non-prescription medicines, vitamins, supplements or herbal remedies; changed diet; cut out alcohol and drugs.

d Those with mental health conditions who didn’t receive enough/any help because they preferred to self-manage.

e Combined social support service use variables including housing, legal aid, job search, youth, family, financial crisis, domestic violence, gambling, alcohol and drug supports.

f Including mental health nurse.

#### Intrapersonal

In rural areas, those with diagnosed 12-month anxiety and/or affective disorders were more likely to self-manage mental health (84.3–86.1%) compared to those without disorders (51.9–54.6%, χ^2^(1) *=* 1021.6, *n* = 3850, *p* < 0.001). For those with disorders, the most identified self-management strategy was making healthy diet choices (49.2–52.0%). For those without disorders, the most common self-management strategy was increasing physical activity (28.9–30.0%).

#### Interpersonal

For those without disorders, ability to get support from friends and family for mental health was the most frequently reported interpersonal strength (96.0–97.0%). For those with disorders, any activities with friends and family in the past 4 weeks was most frequently reported (89.5–91.0%). The proportion of people seeking support from family and friends to self-manage mental health in the past 12 months differed significantly (χ^2^(1) = 723.2, *n* = 3853, *p* < 0.001) for those with disorders (39.4–42.1%) vs for those without disorders (16.5–18.3%).

#### Community

A higher percentage of those without mental health disorders had good, very good, or excellent social connectedness (89.0–89.9%) than those with mental health disorders (60.5–65.1%) (χ^2^(2) *=* 1260.5, *n* = 3,847, *p* < 0.001). Social support services were also utilised for mental health support more for those with disorders (13.3–20.0%) compared to those without (6.6–8.6%) (χ^2^(1) = 195.1, *n* = 3853, *p* < 0.001).

#### Personnel

Those with mental health disorders were significantly more likely (χ^2^(2) = 2320.3, *n* = 3853, *p* < 0.001) to access consultations with any health professional (42.3–50.3%) compared to those without disorders (9.5–11.2%). This trend was reflected in all clinician types.

### Characteristics associated with excellent social connectedness

The results of the logistic regression in Table 4 show that those in rural areas with severe (7.4%, *AOR* = 0.18, *p* < 0.001), and moderate (12.0%, *AOR* = 0.28, *p* < 0.001) disorders were significantly less likely to have excellent social connectedness compared to those with no disorders (32.2%).

**Table 4.** Logistic regression analysis – demographic factors associated with having excellent social connectedness.

Demographics	Rural Australia (inner regional, outer regional, and remote)		
	% with excellent social connectedness^a^ (excellent vs poor, fair, good, very good) (95% CI)	AOR (95% CI)	
	(*n* = 3847)	(*n* = 3557)	*p*
*Age group*			
16–24 years	25.6% (20.6%, 31.5%)	1	
25–34 years	27.4% (21.6%, 34.1%)	0.71 (0.42, 1.19)	0.188
35–44 years	30.7% (26.3%, 35.6%)	0.67 (0.40, 1.14)	0.138
45–54 years	27.0% (24.3%, 34.3%)	0.56 (0.31, 1.02)	0.057
55–64 years	29.3% (24.3%, 34.8%)	0.71 (0.37, 1.37)	0.299
65–85 years	29.5% (26.7%, 32.5%)	0.67 (0.37, 1.20)	0.171
*Sex*			
Male	28.6% (25.3%, 32.1%)	1	
Female	28.4% (25.6%, 31.3%)	1.04 (0.82, 1.32)	0.733
*DSM-IV Severity measure – Australian version*			
No 12-month disorder	32.2% (29.6%, 35.0%)	1	
Mild	24.0% (16.8%, 33.1%)	0.62 (0.38, 1.01)	0.053
Moderate	12.0% (8.2%, 17.2%)	0.28 (0.17, 0.45)	< 0.001
Severe	7.4% (4.6%, 11.8%)	0.18 (0.11, 0.31)	< 0.001
*Highest level of education* ^a^			
Year 10 or less	27.3% (22.7%, 32.5%)	1	
Year 11 or 12	26.6% (22.4%, 31.4%)	1.04 (0.70, 1.54)	0.831
Certificate or diploma	27.2% (24.2%, 30.4%)	1.03 (0.76, 1.40)	0.824
Bachelor’s degree or higher	34.6% (29.6%, 39.9%)	1.31 (0.90, 1.89)	0.156
*Marital status*			
Married or de facto	31.0% (27.9%, 34.3%)	1	
Previously married	28.2% (23.2%, 33.8%)	0.99 (0.64, 1.54)	0.961
Not married	21.6% (17.0%, 27.0%)	0.59 (0.33, 1.06)	0.077
*Socioeconomic status (IRSD)*			
Quintile 1 (lowest)	26.1% (22.3%, 30.2%)	1	
Quintile 2	28.6% (24.1%, 33.6%)	0.93 (0.68, 1.26)	0.619
Quintile 3	29.4% (23.5%, 36.1%)	0.97 (0.69, 1.37)	0.868
Quintile 4	27.5% (23.7%, 31.6%)	0.87 (0.65, 1.14)	0.305
Quintile 5 (highest)	34.4% (29.4%, 39.8%)	1.19 (0.83, 1.72)	0.333
*Household composition*			
Family household	29.1% (26.5%, 31.9%)	1	
Non-family household	25.7% (13.9%, 42.6%)	1.19 (0.48, 2.94)	0.698
Individual household	25.0% (22.3%, 28.0%)	1.06 (0.73, 1.55)	0.755
*Household income* ^a^			
Quintile 1 (lowest)	24.2% (20.0%, 29.0%)	1	
Quintile 2	25.4% (22.2%, 28.9%)	1.03 (0.75, 1.42)	0.836
Quintile 3	29.0% (24.1%, 34.5%)	1.24 (0.84, 1.82)	0.27
Quintile 4	30.5% (25.4%, 36.1%)	1.37% (0.93, 2.03)	0.11
Quintile 5 (highest)	32.5% (28.0%, 37.4%)	1.39 (0.97, 2.01)	0.074

a Missing data from rural participants removed from variable: social connectedness (*n* = 6), highest level of education (*n* = 64), household income (*n* = 231).

As area-level socioeconomic disadvantage decreased and household income increased, a trend suggested higher levels of excellent social connectedness; however, these trends were not statistically significant. Once controlling for mental disorder severity level, excellent social connectedness was not significantly associated with age, sex, higher education level, marriage or de facto relationships, higher income and socioeconomic status, or living in a family household.

## Discussion

The RSSF (Luebbe et al., 2024) was used to organise and interpret the findings of this study. The Framework provides a lens to identify which factors (e.g. individuals, services, community organisations) foster or sustain strengths. Its application supports targeted policy and service responses by allowing decision makers to identify key areas of improvement or prioritisation. The NSMHW 2020–2022 (ABS, 2023a) survey questions reflected strengths beneficial to mental health across intrapersonal, interpersonal, community and personnel levels of the Framework (Luebbe et al., 2024). However, it largely missed strengths related to culture, nature and lay community members, rural norms and standards (e.g. flexible and innovative service delivery, interdisciplinary collaboration). In addition, some strengths were identified briefly or indirectly and were only asked of a particular group (i.e. spirituality questions asked only of those who did not receive enough support for their mental health because they preferred to self-manage). Question framing limited insight into *why* rural Australians seek support from specific providers. To overcome these issues, future national surveys could incorporate broader strengths-based measures, drawing on examples such as Australia’s National Aboriginal and Torres Strait Islander Social Survey (NATSISS) (ABS, 2016) which includes cultural practices, community support and social networks. Additional questions about service use and help-seeking could provide more context, such as the importance of having trust in a provider, whether providers had rural lived experience and community involvement, or lived experience of mental illness, and whether these attributes impacted reasons for seeking help.

Physical activity and healthy diet choices were commonly identified self-management strategies across rural areas. Both exercise and diet are widely promoted (Catford, 2000; Wise, 2008) and well-regarded (Lloyd and Sullivan, 2003) for mental health. They may be less stigmatised than other strategies like mindfulness, which can be used less frequently (Remskar et al., 2022). Family and friend support also emerged as a key strength, reinforcing the role of close social networks. These individuals often serve as informal first-responders and could benefit from resources that enhance their capacity to support others (Diminic et al., 2019; Leggatt, 2007; Ohaeri, 2003). Few differences were found across rurality levels, though remote populations reported lower use of diet-related strategies, likely due to limited access to affordable and nutritious food (Whelan et al., 2018). Addressing food insecurity could indirectly improve mental health outcomes (Ejiohuo et al., 2024). Individuals with affective or anxiety disorders reported higher self-management and professional service use, suggesting that self-management complements rather than replacing formal care. Enhancing mental health literacy, promoting early intervention and destigmatising mental illness remain important issues, especially in rural contexts where self-reliance and resourcefulness is common (Nelson et al., 2022; Page-Carruth et al., 2014) and professional services are less accessible.

Excellent self-reported social connectedness was positively associated with better mental health, consistent with prior research on social capital; connected rural communities have improved mental health (Galloway, 2013; Wickramaratne et al., 2022). Having a strong sense of belonging and community support buffers distress following environmental disasters such as floods and droughts (Matthews et al., 2020; Stain et al., 2008). Leveraging existing community and mateship in rural Australia may offer relevant strategies to support mental well-being, particularly those with more severe mental illness. To embed these findings into service planning, data collection and models should shift away from deficit-based approaches and recognise social connectedness as a valuable resource, integrating strengths into rural mental health care.

### Limitations

This study was constrained by data limitations which may have affected scope and depth of analyses. The survey sampling excluded discrete Aboriginal and Torres Strait Islander populations and very remote communities. These groups are often under-researched and may have specific strengths that warrant further research. The NSMHW also uses the ARIA geographic classification tool, which is not as nuanced as the Modified Monash Model (MMM) (Australian Government Department of Health, 2023), that makes greater distinctions across more levels of rurality. However, current remoteness classification tools provide limited information about place-based context and can misconstrue findings and falsely imply that areas within the same rurality classification must be similar. Drawing on place-based perspectives, differences within regional areas are recognised, and the unique context of each community should be considered (Butterworth et al., 2024). This study was unable to explore this variation. In addition, this study focused on anxiety and affective disorders (see Methods), with alcohol and drug use disorders outside scope. The relationship between health and social connectedness may be bidirectional, as better mental health may enable participation, while depressive symptoms (e.g. reduced energy and anhedonia) may limit social engagement or contribute to social withdrawal. While the response rate (52%) is comparable to similar national surveys, non-response bias remains possible and should be considered when interpreting results. As with all structured diagnostic interviews, the WMH-CIDI is subject to limitations including reliance on lay interviewers and restricted probing of symptom nuance.

## Conclusion

This study has generated a snapshot of how rural populations identify and use strengths for their mental well-being, through a national mental health survey. The NSMHW identified existing assets and resources within rural individuals, intrapersonal relationships, community connections, and health care providers. Despite survey question constraints and the study not being explicitly designed to capture strengths, many positive strategies were identified across the RSSF and used throughout rural Australia. While rural areas are frequently described in terms of service, system and workforce shortages, rural people draw on a range of management strategies and personal and community connections. Where strengths differed between mental well-being and rurality, results suggest the need to focus efforts on specific rural populations. Areas requiring a particular focus include improving healthy diet self-management strategies in remote populations and supporting social connections for people with more severe mental illness. While some strengths differed minimally by rurality, it is recommended that local context, rather than geographical classification, may be more indicative of how and/or where strengths are useful and should be harnessed. Future research should explore strengths-based approaches that collect data on rural assets, resources, and protective factors for mental health, as well as qualitative data on what promotes and maintains well-being.
